# Resistance Induction and Direct Antifungal Activity of Some Monoterpenes against *Rhizoctonia solani,* the Causal of Root Rot in Common Bean

**DOI:** 10.3390/life12071040

**Published:** 2022-07-12

**Authors:** Aly Derbalah, Asmaa Mohamed Shebl, Samah Fawzy Elgobashy, Abdelmonim Ali Ahmad, Noha Eldesoky Ramadan, Said I. Behiry, Ahmed Abdelkhalek, Muhammad Hamzah Saleem, Abdulaziz A. Al-Askar, Muhammad Kamran, Mohsen Mohamed Elsharkawy

**Affiliations:** 1 Pesticides Chemistry and Toxicology Department, Faculty of Agriculture, Kafrelsheikh University, Kafr El Sheikh 33516, Egypt; ali.derbala@agr.kfs.edu.eg; 2 Plant Pathology Research Institute, Agriculture Research Center, Giza 12619, Egypt; asmaashebl159@gmail.com (A.M.S.); samahelgobashy@gmail.com (S.F.E.); hm_na10@yahoo.com (N.E.R.); 3 Department of Plant Pathology, Faculty of Agriculture, Minia University, El-Minia 61519, Egypt; abdelmonim.ali@mu.edu.eg; 4 Agricultural Botany Department, Faculty of Agriculture (Saba Basha), Alexandria University, Alexandria 21531, Egypt; said.behiry@alexu.edu.eg; 5 Plant Protection and Biomolecular Diagnosis Department, ALCRI, City of Scientific Research and Technological Applications, New Borg El-Arab City, Alexandria 21934, Egypt; aabdelkhalek@srtacity.sci.eg; 6 MOA Key Laboratory of Crop Ecophysiology and Farming System Core in the Middle Reaches of the Yangtze River, College of Plant Science and Technology, Huazhong Agricultural University, Wuhan 430070, China; saleemhamza312@webmail.hzau.edu.cn; 7 Department of Botany and Microbiology, College of Science, King Saud University, P.O. Box 2455, Riyadh 11451, Saudi Arabia; aalaskara@ksu.edu.sa; 8 School of Agriculture, Food and Wine, The University of Adelaide, Adelaide, SA 5005, Australia; muhammad.kamran@adelaide.edu.au; 9 Department of Agricultural Botany, Faculty of Agriculture, Kafrelsheikh University, Kafr El Sheikh 33516, Egypt

**Keywords:** common bean, monoterpenes, root rot, resistance induction, pathogenesis related genes

## Abstract

This study was conducted to evaluate eco-friendly control agents (carvone, cuminaldehyde, and linalool) against *Rhizoctonia solani,* which causes root rot disease either by induction of defense response or direct antifungal activity. The induction of resistance was examined by detecting the transcription of defense genes and the effect of the tested control agents on the growth and the yield of common bean plants. The growth of *R. solani* was significantly inhibited after treatment with the tested compounds compared to the untreated control under laboratory conditions. The disease severity of root rot was decreased in common bean plants treated with the tested compounds compared to untreated control plants under greenhouse conditions. Common bean plants treated with the tested control agents expressed defense genes (*Phenylalanine ammonia lyase* and *β-1,3-Glucanase*) involved in jasmonic acid (JA) and salicylic acid (SA) signaling pathways with 2–5 fold higher than the control. Treatment of common beans with the tested control agents and fungicide significantly improved the growth and yield characteristics of common bean. Therefore, the use of monoterpenes could be a novel strategy to control this pathogen and consider the first report.

## 1. Introduction

The common bean (*Phaseolus vulgaris* L.) is a universal legume that is an essential source of protein for humans, particularly for the poor [1]. Vitamins, minerals, and other nutrients are abundant in this food [2]. The common bean is an important commercial crop in Egypt, with an annual dry bean production of 98,132 tons, and the harvest area is 39,665 hectares according to the General Authority for Statistics [3]. Fungi are the primary cause of yield losses in the common bean, which is susceptible to a wide range of diseases [4]. The fungi cause the majority of common bean infectious diseases including damping off caused by *R. solani* [5]. There are a variety of plant diseases caused by the soil-borne pathogens, for example *R. solani*, which affects a broad range of plant species, including bean [6,7]. *R. solani* and *Macrophomina phaseolina* are two of the most frequent diseases in Egypt, causing significant losses in yield production [8].

To manage fungal diseases, a variety of approaches are used. The use of chemical fungicides is the most popular method used by the growers. Environmental contamination and the creation of novel fungal strains are possible consequences of excessive chemical fungicide usage [9]. Hence, there has been steady interest in research regarding the potential use of plant secondary metabolites for disease control [10,11,12]. Phytochemicals, such as terpenoids, phenols, alkaloids, and glucosides, have long been recognized as a class of chemical substances found in plants that may be used to control pests [13,14,15,16]. Recently, plant extracts have gained significant interest as alternative options to synthetic fungicides and efforts have been made to use these extracts in plant disease control strategies [10,11,12].

In terms of pest and disease management, monoterpene-rich essential oils are the most effective and least toxic option [13,14]. Plant protection might benefit from the unique qualities of monoterpenes, such as low vapor pressure, their lipophilicity, and minimal toxicity to mammals. Monoterpenes showed numerous biological activities as insecticides, fungicides, herbicides, and bactericides [13,14,15,16,17,18,19,20,21]. Two postharvest pathogens, *Monilinia fructicola* and *Botrytis cinerea*, were used to evaluate the antifungal activity of 22 phenylpropenes and monoterpenes [17]. Furthermore, 31 plant-pathogenic fungi were used to test the antifungal effects of oxygenated monoterpenes [18]. The mycelial growth of certain fungi was inhibited by some of the monoterpenes that were studied. *Colletotrichum gloeosporioides*, *Colletotrichum musae*, and *Fusarium subglutinans* f. sp. *ananase* all recorded significant growth inhibition when exposed to l-carvone [21].

Hence, this study was intended to evaluate the antifungal activity of some compounds from plant origin (carvone, cuminaldehyde and linalool) against *R. solani* under laboratory conditions, to determine the extent of their ability to control root rot disease in common bean plants under greenhouse and field conditions, to determine the induction of the defense response through transcription of defense genes using qRT-PCR, and lastly to examine the effect of tested control agents on some growth and yield characteristics of common bean.

## 2. Materials and Methods

### 2.1. Chemicals

The examined monoterpenes (cuminaldehyde, linalool, and carvone) with a purity of 99% were obtained from Sigma Aldrich, St. Louis, MO, USA. A fungicide Toclophos-methyl + Thiram with a trade name of Rhizolex 50 WP was purchased from Sumitomo chemical company, Japan.

### 2.2. Isolation, Purification, and Identification of Pathogenic Fungus

Pathogenic isolate of *R. solani* was isolated from naturally infected common bean plants, showing root rot symptoms. The isolated fungus was purified using hyphal tip technique and identified based on the morphology and microscopic characteristics. Identification was confirmed in Mycological Research and Disease Survey Department, Plant Pathology Research Institute (PPRI), ARC, Giza, Egypt. The pathogen was identified according to Sneh et al. [22]. The pathogenic isolate was maintained on potato dextrose agar medium (PDA) at 4 ± 1 °C [23].

### 2.3. In Vitro Antifungal Action of the Used Monoterpenes against R. solani

The fungal isolate was cultured onto PDA medium for 7 d, then plugs (5 mm) was taken and re-cultured again onto PDA plate (9 cm) poisoned with monoterpenes (cuminaldehyde, linalool, and carvone) and fungicide with different concentrations of 10, 20, 50, and 100 µg/mL. Five plates were served as replicates for each treatment. Plugs of *R*. *solani* grown onto PDA were used as control. At 25 °C at 7 d, the Petri dishes were incubated. To analyze the antifungal effects of monoterpenes, such as a reduction in pathogenic fungus mycelia development, the following formula was developed:

Antifungal effect = (A − B/A) × 100

where, A: The diameter of mycelia growth of pathogenic fungus in the control and B: The diameter of mycelial growth of pathogenic fungus with cuminaldehyde, linalool, and carvone. The experiment was repeated three times. The light microscope (Leica DM1000) was used to analyze and photograph the mechanism of action of monoterpenes and fungicide on *R. solani*.

Scanning electron microscopy (SEM) has been deployed to assess the impact of monoterpenes on the development of *R. solani* [24]. The samples were submerged for fixation in a solution (2.5% buffered glutaraldehyde + 2% paraformaldehyde in 0.1 M sodium phosphate buffer pH 7.4). After an overnight incubation at 4 °C, the tissues were washed three times with a 0.1 M sodium phosphate buffer and a 0.1 M sucrose solution before being used. At pH 7.4 and 2 % salt, the tissues were post-fixed for 90 min. In order to wash the samples, sodium phosphate buffer pH 7.4 (0.1 M) was employed for three cycles. A series of ethanol dilutions were used to dehydrate samples. For the SEM analysis, materials were placed in critical point drying, and specimens were covered with gold palladium membranes.

### 2.4. Efficacy of the Tested Monoterpenes against Root Rot of Common Bean under Greenhouse Conditions

Common bean seeds of cv. Paulista were procured from the Horticulture Research Institute, ARC, Giza, Egypt. For this experiment, sand clay soil was sterilized using formalin with a concentration of 5% and air dried for 7 days [25]. The sterilized soil was then transferred to a 25 cm diameter pot, each containing 3 kg soil. A dry artificial inoculation of the soil was carried out with *R. solani* at a rate of 1.5% using the hull rice cultivation (*w*/*w*). Common bean seeds were soaked in the solutions of examined monoterpenes (100 µg/mL) and the recommended fungicide (2 g/kg seeds) for 30 min. The seeds of control treatment were soaked with distilled water only for the same time. After soaking, the treated bean seeds were germinated on filter papers at 24 °C for 48 h in the dark, as described by Wen et al. [26]. Common bean seeds were grown (10 seeds/pot). Each treatment was represented by three replicates. 

The method described by Khalifa [27] was used to calculate the pre- and post-emergence damping-off percentages, as well as the healthy survival percentages, in each treatment at 15, 30, and 45 d after sowing.

$$Pre\;emergence\;\left(\%\right)=\frac{Number\;of\;non\;germinated\;seeds}{Total\;number\;of\;sown\;seeds}\times 100\;$$

$$Post\;emergence\;\left(\%\right)=\frac{Number\;of\;dead\;plants\;after\;germination}{Total\;number\;of\;sown\;seeds}\times 100$$

$$Survival\;plant\;\left(\%\right)=\frac{Number\;of\;survival\;plant}{Total\;number\;of\;sown\;seeds\;}\times 100$$

Disease incidence percentage was calculated as following:

$$\mathrm{Disease}\;\mathrm{incidence}\;(\mathrm{DI}\;\%)=\;\frac{\mathrm{Number}\;\mathrm{of}\;\mathrm{infected}\;\mathrm{plants}}{\mathrm{Total}\;\mathrm{plants}\;\mathrm{in}\;\mathrm{treatment}}\;\times 100$$

### 2.5. Efficacy of the Tested Monoterpenes against Root Rot of Common Bean under Field Conditions

Fields with a history of damping off disease in Kafrelsheikh and Gharbia governorates were utilized to evaluate the effects cuminaldehyde, linalool, and carvone, in different blocks on root rot disease. The efficacy of the tested monoterpenes (100 µg/mL) was compared with a standard fungicide (Tolclofos-methyl+ thiram). The common bean seeds were soaked in the solutions of the examined monoterpenes and the recommended fungicide with the same concentration as mentioned before in greenhouse experiment. The field experiment (25 plots) was conducted in a completely randomized block design with five replicates in each plot. Each plot had a surface area of 3 × 4 m^2^ and four rows that were 4 m in length and 75 cm wide. Seven days before planting, the soil was irrigated, and each hole was sown with two treated bean seeds (cv. Karnak) spaced 20 cm apart. The pre- and post-emergence damping-off percentages were determined as explained in the greenhouse experiment. Evaluation of the efficacy of each treatment was computed according to the following formula adopted by Rewal and Jhooty [28].

Efficacy = (DI in control − DI in treatment/DI in control) × 100

### 2.6. Efficacy of the Tested Monoterpenes on Growth and Yield Parameters of Common Bean under Field Conditions

For each treatment and the control plants (infected with the pathogen) under field conditions in both Kafrelshiekh and Gharbia governorates, random samples of 10 bean plants were taken 60 d after planting. Plant growth indicators such as plant height, number of branches per plant, and fresh and dried plant weight were measured. The number of pods per plant, average pod weight, production per hectare, and percent rate of growth were also recorded as yield characteristics. The percentage of yield increase was calculated using the following equation.

Yield increase (%) = (Yield of treatment − Yield of control/Yield of control) × 100

### 2.7. Effect of the Tested Monoterpenes on Defense Enzymes

In bean plants under greenhouse conditions, the effects of cuminaldehyde, linalool, and carvone on the activity of peroxidase, and polyphenol oxidase enzymes involved in plant defense against pathogen infection were investigated.

For enzyme tests, 3 mL of 50 mM TRIS buffer (pH 7.8) containing 1 mM EDTA-Na_2_ and 7.5% polyvinylpyrrolidone was homogenized at 0–4 °C with 0.5 g of leaf materials collected after 15 d of treatments. Total enzyme activity in the supernatant were determined by spectrophotometer after centrifugation (12,000 rpm for 20 min at 4 °C) of the homogenates. The UV-160A spectrophotometer (Shimadzu, Japan) was used for all measurements at 25 °C. Three different sets of enzymes were used in the experiment. In a typical approach provided by Hammerschmidt et al. [29], the activity of the peroxidase (POX) was directly measured from the crude enzyme extract. For 3 min, changes in absorbance at 470 nm were monitored every 30 s. As a measure of enzyme activity, the change in absorbance min^−1^ g^−1^ fresh weight was calculated. The activity of polyphenoloxidase (PPO) was measured as stated in Mayer et al. [30]. For 3 min, the absorbance at 495 nm was measured at 30 s intervals. The change in absorbance min^−1^ g^−1^ fresh weight was used to measure enzyme activity. Activity of catalase was reported as mmol min^−1^ g^−1^ of fresh weight [31].

### 2.8. Analysis of Defense Related Genes Expression in Common Bean Plants Treated with the Tested Monoterpenes

Leaves from all monoterpene-treated plants, as well as the control (non-treated and infected plants), under greenhouse conditions were collected after one and two weeks of treatments. A mortar and pestle were used to grind the leaf tissue of each treatment with liquid nitrogen into a fine powder. Utilizing an RNA purification kit, RNA was extracted from bean leaves (Thermo Scientific, Waltham, MA, USA, Fermentas, # K0731). Samples were homogenized in Lysis Buffer (300 μL) with mercaptoethanol. The lysate was then combined with ethanol and put on a purification column. Vortexing for 10 s was used. Diluted Proteinase K (600 μL) was added, mixed completely, and incubated at 20 °C for 10 min. After 5 min at 12,000× *g*, supernatant was transferred to a fresh RNase-free tube. Ethanol (100%, 450 μL) was added. Lysate (700 μL) was put to GeneJETTM RNA Purification Column in a collecting tube. The column was centrifuged for 1 min at 10,000 rpm, then the flow through was discarded and the purification column was inserted back into the collecting tube. The collection tube holding the flow-through solution was discarded and the column was put in a fresh 2 mL collection tube. Wash Buffer I (700) was added to the Column and centrifuged for 1 min at 10,000 rpm. The flow through was discarded and the purification column was put back into the collecting tube. Wash Buffer II (600 μL) was added to the Column and centrifuged (1 min at 10,000 rpm). Nuclease-free water (100 μL) was poured to the middle of the column membrane and centrifuged (1 min at 10,000 rpm) to elute RNA. The purified RNA was utilized for downstream applications and stored at −80 °C until use. DNase was treated with the RNase-Free DNase Set (Qiagen), according to the manufacturer’s recommendations. Reverse Transcription Kit (Thermo Scientific, Fermentas, # EP0451) was used to make complementary DNA (cDNA). In a sterile, nuclease-free tube, 5 µg of template RNA, 0.5 µg Oligo dT, and DEPC-treated water were added and completed to 12.5 μL. Reaction Buffer (4 μL of 5x), 0.5 μg RiboLock RNase Inhibitor, 1 μL RevertAidTM H Minus Reverse Transcriptase, and 2 μL dNTP Mix were added to the nuclease-free tube. After mixing, incubation at 42 °C for 60 min was done. After incubation for 10 min at 70 °C, the process was stopped. *Actin* was employed as an internal reference to quantify the expression of the two target genes *Phenylalanine ammonia lyase (PAL*) and *β-1,3-Glucanase (GLUC)* using quantitative RT-PCR (Step One Plus™, Applied Biosystems, Foster City, CA, USA) with SYBR Green according to the manufacturer’s methodology (Thermo Scientific, Waltham, MA, USA, # K0221) (Table 1) [26]. ^2-∆∆^Ct approach was used to normalize the target gene’s critical threshold (Ct) amounts with those of the housekeeping gene (*Actin*) [32].

### 2.9. Data Analysis

Regression analysis is an established tool for pinpointing the specific factors that contribute to an investigation’s findings. It provides conclusive information about the relative importance of different variables and their interactions. For analysis of variance (ANOVA) of obtained data, XLSTAT PRO statistical analysis software (Addinsoft) was used. Fisher’s least significant difference (LSD) test was used to separate the means of each treatment. All analyses were performed at a significance value of *p <* 0.05.

## 3. Results

### 3.1. Antifungal Activity of Monoterpenes against R. solani under Laboratory Conditions

**Figure 1:**
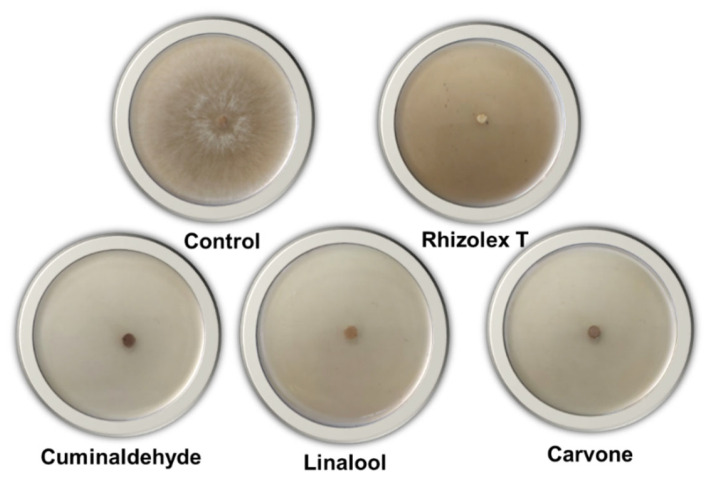
Antifungal effects of monoterpenes on radial growth of *R. solani* (7 days after incubation). Treatments are control, fungicide (Rhizolex T), cuminaldehyde (100 µg/mL), linalool (100 µg/mL), carvone (100 µg/mL).

The tested isolate of *R. solani* was allowed to grow in vitro on agar medium supplemented with 10, 20, 50, and 100 µg/mL of the examined monoterpenes as well fungicide (Figure 1). Table 2 shows the growth inhibition of the tested monoterpenes against *R. solani* in laboratory conditions. In comparison to the untreated control, examined monoterpenes and fungicide at various concentration levels, potentially suppressed the development of *R. solani*. As shown in Table 2, the highest percentage growth inhibition of *R. solani* was achieved at the highest concentration (100 µg/mL). Regression analysis showed that the efficacy of examined monoterpenes was concentration dependent, with a positive correlation between efficacy and concentration. The highest growth inhibition of *R. solani* was recorded for fungicide followed by carvone, cuminaldehyde, and linalool, respectively. 

**Figure 2:**
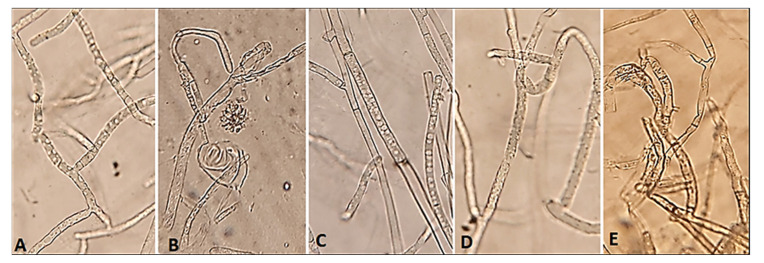
Light microscopy examination of monoterpenes antifungal effects against *R. solani*, (**A**) control, (**B**) fungicide (Rhizolex T), (**C**) cuminaldehyde (100 µg/mL), (**D**) linalool (100 µg/mL), (**E**) carvone (100 µg/mL).

**Figure 3:**
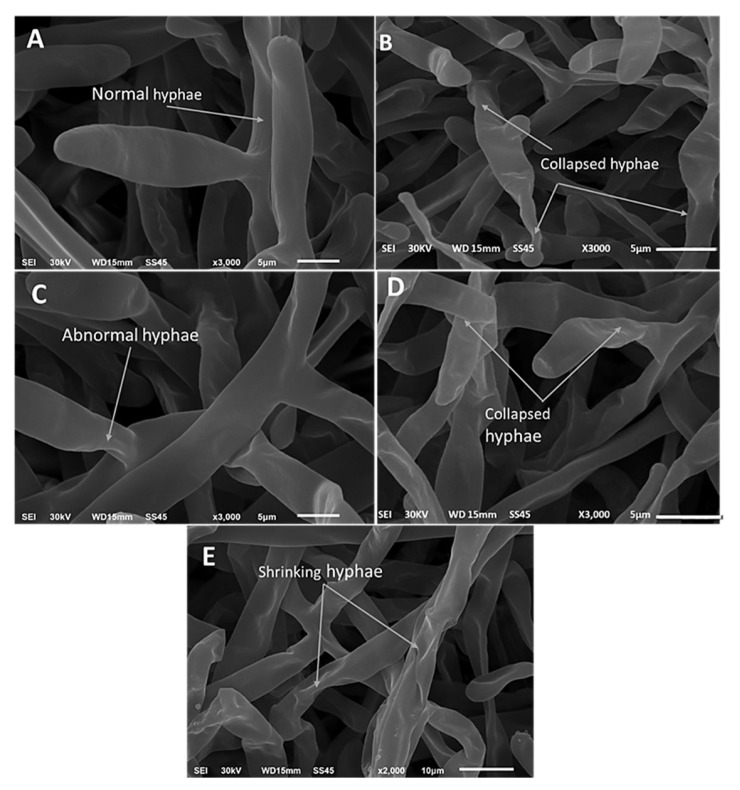
Scanning Electron Microscopy (SEM) of monoterpenes antifungal effects against *R. solani*, (**A**) control, (**B**) (Rhizolex T), (**C**) cuminaldehyde (conc. 100 µg/mL), (**D**) linalool (conc. 100 µg/mL), (**E**) carvone (conc. 100 µg/mL).

The light microscope examination showed a complete fungal growth of *R. solani* in the control (Figure 2A). Full coalescence was developed from the mycelia in their ideal form in the control. However, the treatments with the fungicide or monoterpenes showed morphological anomaly and lysis of *R. solani* (Figure 2B–E). Typical morphological characteristics of *R. solani* was observed using SEM in the control (Figure 3A). In contrast, lysis and hyphae shrinking and collapsing were observed in cultures of *R. solani* treated with monoterpenes and the fungicide (Figure 3B–E).

### 3.2. Systemic Protection against Root Rot Disease in Common Bean under Greenhouse Conditions

The results in Table 3 showed that post-emergence damping off and disease severity were significantly reduced in common bean plants treated with monoterpenes and the recommended fungicide compared to untreated control. The effects of monoterpenes were a little lower than the recommended fungicide in the reduction of post-emergence damping off and disease incidence of treated common bean plants in spite of the concentration of fungicide being much higher than that of monoterpenes. The reduction in the incidence of root rot disease in common bean treated by cuminaldehyde and tolclofos-methyl + thiram was higher than carvone and linalool treatments.

### 3.3. Systemic Protection against Root Rot Disease in Common Bean under Filed Conditions

Table 4 shows that in common bean plants treated with the tested monoterpenes and the recommended fungicide, both pre- and post-emergence damping off, as well as disease incidence, were significantly reduced when compared to untreated controls whether in Gharbia or Kafr Elsheikh governorates. Despite the fact that the fungicide concentration was much higher than that of monoterpenes, monoterpenes had a slightly lower effect than the recommended fungicide in reducing pre- and post-emergence damping off as well as disease incidence in treated common bean plants. Based on the reduction in damping-off and disease incidence of root rot disease in treated common bean, carvone was the most effective monoterpene, followed by cuminaldehyde and linalool, in that order, whether in Gharbia or Kafr Elsheikh governorates.

### 3.4. Effect of Monoterpenes on Bean Growth and Yield

The effect of the used monoterpenes and the fungicide on some growth and production characteristics of the bean plants under field conditions was clarified in Table 5 and Table 6. All vegetative traits were found to be significantly increased in treated bean plants compared to untreated control, whether it was in Gharbia Governorate or Kafrelsheikh as shown in Table 4 and Table 5. Fungicide, followed by carvone, cuminaldehyde, and linalool, were the most effective treatments on the growth or yield characteristics of bean plants, whether in Gharbia or Kafr Elsheikh governorates.

### 3.5. Effect of Monoterpenes of Defense Enzymes Activities

Establishing resistance in plants is facilitated by plant defense enzymes (POX, PPO, and CAT). The activities of POX, PPO, and CAT in bean treated with monoterpenes against root rot disease were evaluated in order to identify any possible defense enzymes implicated in resistance initiation. The activities of POX, PPO, and CAT rose dramatically in bean leaves treated with the examined monoterpenes, specially carvone and cuminaldehyde followed linalool and the fungicide, respectively (Table 7). Meanwhile, the activity of PPO significantly increased after treatment with carvone more than other treatments. 

### 3.6. Effect of Monoterpenes on the Expression of Defense-Related Genes in Treated Common Bean Plants

**Figure 4:**
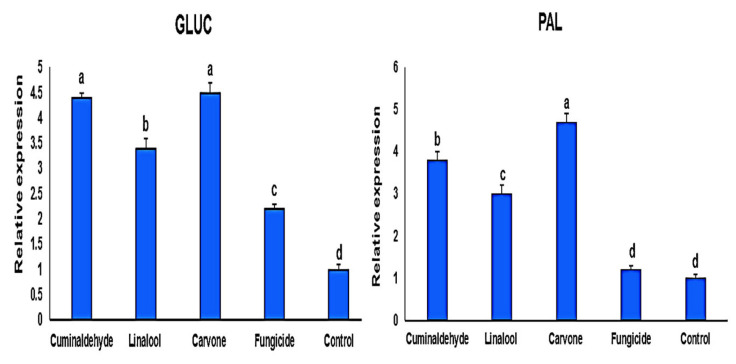
Real-time quantitative reverse transcription-polymerase chain reaction (qRT-PCR) efficiencies for the defense-associated genes *GLUC*, and *PAL* at 1 week after treatments. The different letters represent significant differences.

**Figure 5:**
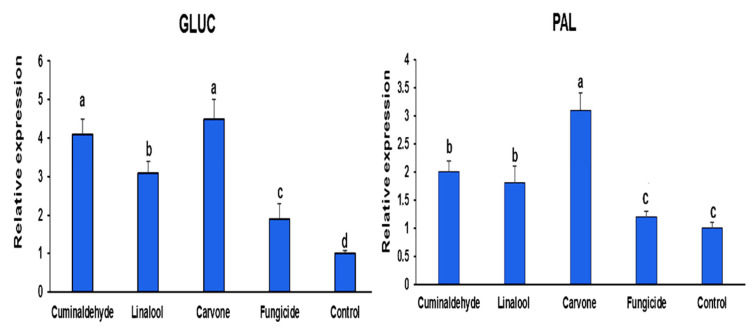
Real-time quantitative reverse transcription-polymerase chain reaction (qRT-PCR) efficiencies for the defense-associated genes *GLUC* and *PAL* at 2 weeks after treatments. The different letters represent significant differences.

The expression of pathogenesis-related genes (*GLUC* and *PAL*) was significantly increased in common bean plants treated with monoterpenes after one and two weeks of treatments (Figure 4 and Figure 5), while the targeted responsive genes in untreated plants showed no stimulated expression of defense genes. Protected and infected seedlings showed a significant up-regulation (*p <* 0.05) of transcript levels in leaf tissues for *GLUC* and *PAL* genes compared with those that are infected and not protected plants. Moreover, carvone treatment showed the highest expression levels of pathogenesis-related genes followed by cuminaldehyde and linalool, respectively. This increase ranged between two- and five-folds depending on the tissue and the gene analyzed (Figure 4). Of interest, the relative expression ratio of *GLUC* and *PAL* genes in protected and infected tissues was higher in the first week than the second week after treatments (Figure 4 and Figure 5).

## 4. Discussion

Synthetic chemical fungicides currently in use are harmful to public health. Hence, biocontrol agents and plant secondary metabolites, such as essential oils and other volatile aromatic products, have been extensively studied to evaluate their efficacy against plant pathogenic fungi [19,20,21]. This study evaluated the antifungal activity of some bioactive components, carvone, cuminaldehyde, and linalool, against *R. solani*. The results showed that the tested monoterpenes inhibited the development of *R. solani* at different concentrations and showed fungicidal activity under laboratory conditions. This is in agreement with some studies that documented the antifungal activity of some monoterpenes and phenylpropenes against plant pathogenic fungi. For example, Kordali et al. [18] evaluated the antifungal activities of some oxygenated monoterpenes, including camphor, carvone, 1,8-cineole, fenchone, geraniol, linalool, and menthol against 31 plant pathogen fungi. They stated that some monoterpenes had potent inhibitory effects against most of the tested fungal species. Thymol was also approved to completely inhibit the fungal growth of 17 types of plant pathogenic fungi, including *R. Solani* and *Fusarium oxysporum* [19]. In addition, carvone was shown to control potato sprouting and showed effective antifungal activity against other potato storage diseases caused by *F. sulphureum*, *Phoma exigua* var. *foveate* and *Helminthosporium solani* [20]. In the same way, Garcia et al. [21] demonstrated that carvone strongly inhibited the growth of post-harvest fungi *Colletotrichum musae*, *C. gloeosporioides*, and *F. subglutinans* f. sp. *ananas*. Different mechanisms of action of monoterpenes or Eos on fungal species have been described, such as ruptured cell wall and membrane disruption, inhibition of chitin synthesis, ROS accumulation, mitochondrial dysfunction, and inhibition of some specific enzyme activities [33,34,35].

As part of the objectives of this study, the ability of three monoterpenes, carvone, cuminaldehyde, and linalool to control root rot pathogens under laboratory and greenhouse conditions was evaluated. The results in this study indicated that the investigated monoterpenes showed a high potency to reduce root rot disease severity in common bean under greenhouse conditions. As previously reported by other authors, foliar application of plant extracts or Eos could be able to significantly reduce the severity of phytopathogens caused by soil-borne fungi, such as *R. solani* [36]. Research into the mechanisms of disease inhibition by plant extracts and Eos have indicated that their active components may act directly on the pathogen [37] or by activating defense responses in host plants, leading to a reduction in disease progression [38].

In greenhouse experiment conducted on common bean, treatment with monoterpenes reduced the severity of root rot disease. These results position monoterpenes as a promising resistance elicitor against root rot in common bean plants. Treatment with monoterpenes activated the expression of pathogenesis-related (PR) proteins. Also, it regulates the activities of various enzymes such as, peroxidase (POD) and polyphenoloxidase (PPO), which are the main components of induced plant defense against biotic and abiotic stresses [39]. The significant increase in the spatial expression of transcript levels of *PAL* and *GLUC* in bean seedlings due to monoterpenes treatments and *R. solani* infection is a strong indication that *R. solani* stimulated a systemic accumulation of PR proteins [40], and production of POX and PPO, two key enzymes in defense response [39]. This phenomenon implies, therefore, the existence of a signal that spreads systematically from the hypocotyls to the rest of the plant. Many studies have indicated that SA, a natural phenolic compound, is an important signaling factor in the induction of SAR [39]. 

This study presents a novel use of selected bioactive monoterpenes to control the pathogen, *R. solani*, in the production of common bean. This technology is most effective in production systems with a rapid turnover rate because most of the essential oil components are volatile and degrade rapidly. The continuous development of this technology will serve the systems of organic or sustainable cropping systems where it is not undesirable to introduce chemical pesticides in the control process [41]. EOs marketed as alternatives to fungicides to control plant pathogenic fungi, post-harvest fungi, and food preservation [36,37]. It is important in this regard to search for environmentally friendly and harmless control agents at low doses to reduce the economic losses caused by these plant pathogens. 

The results of our study showed that the treatment with examined monoterpenes improved the growth and yield characters of common bean plants. This is in agreement with what was mentioned by Abdel-Mawgoud et al. [42] that the application of the plant extract improved the various growth indicators of watermelon plants. In the same context, Shehata et al. [43] on celeriac plants, Fawzy et al. [44] on Chinese garlic plants, and Hernández et al. [45] on tomatoes, found that the application of botanical extract that contains phenolic compounds such as the examined monoterpenes in the present study as a foliar spray gave the highest values for vegetative growth. Also, the foliar application of licorice extract had the highest stimulation effect on plant growth characters. The stimulating effect on the growth behavior of bean plants treated with licorice extract can be explained because this extract contains a high concentration of natural chemical compounds such as; phenolic compounds that are necessary for plant physiology due to their contribution to plant morphology and structure, and they are also involved in plant growth and reproductive process [46].

The positive effects of the examined monoterpenes on growth parameters and common bean production were observed. Thus, the application of environmentally friendly monoterpenes can be considered as a good production strategy to obtain high yields of nutritious vegetables with less impact on the environment [47]. In the same context, bio stimulants containing essential oils such as monoterpenes used in this study are able to enhance vegetative growth, mineral nutrient absorption, and improve productivity of many plants [48,49,50].

The negative effects of synthetic chemicals, such as fungicide residues in food, fungal strain resistance, and environmental pollution, might be mitigated with the introduction of effective natural pesticides. In this respect, natural substances such as carvone, cuminaldehyde, and linalool may be employed as non-toxic alternatives for controlling plant diseases caused by plant pathogenic fungus.

## 5. Conclusions

Based on the results obtained from this study, the tested monoterpenes had fungicidal activity against *R. solani* under laboratory conditions. The tested monoterpenes reduced the disease incidence of the pathogen on normal beans under greenhouse and field conditions. The disease incidence was reduced either by the direct antifungal effect of these compounds or by improving plant resistance to the diseased pathogen. It also led to improving the growth and production characteristics of the treated plants. For this, the examined monoterpenes can be considered as new alternative strategies for disease management.

## Figures and Tables

**Table 1:** Common bean sequences used for primer design for RT-PCR analysis.

Primer Name	Forward Primer (5′–3′)	Reverse Primer (5′–3′)	Accession Number	Product Size (pb)
GLUC	GCTGTAAGGGCTCAAGGCCTC	CCAAGTACACACGTGCGTTGTC	X53129	427
PAL	AAGCCATGTCCAAAGTGCTG	GAGTTCTCCGTTGCCACCT	M11939	240
ACTIN	CACCGAGGCACCGCTTAATC	CGGCCACTAGCGTAAAGGGAA	AB067722	126

**Table 2:** Radial growth and inhibition percentage of the tested treatments against *R. solani* in vitro with regression equation.

Treatments	Concentration (µg/mL)	Linear Growth (cm)	Growth Inhibition %	Regression Equation	R²
Cuminaldehyde	10	7.2 ± 1.3 ^b^	20	y = 0.8776x + 10.51	0.99
	20	6.3 ± 1.2 ^b^	30		
	50	4.5 ± 0.9 ^c^	50		
	100	0.0 ± 0.0 ^f^	100		
Linalool	10	7.4 ± 1.2 ^b^	18	y = 0.9276x + 9.0102	0.99
	20	6.8 ± 1.1 ^b^	25		
	50	3.3 ± 0.7 ^d^	60		
	100	0.0 ± 0.0 ^f^	100		
Carvone	10	4.9 ± 0.8 ^c^	45	y = 0.5969x + 41.888	0.99
	20	4.1 ± 0.7 ^c^	55		
	50	2.3 ± 0.7 ^e^	75		
	100	0.0 ± 0.0 ^f^	100		
Rhizolex T	55	4.1 ± 0.9 ^c^	55	y = 0.4796x + 53.418	0.98
	65	3.2 ± 79 ^d^	65		
	80	1.8 ± 0.6 ^e^	80		
	100	0.0 ± 0.0 ^f^	100		
Control	0.00	9.0 ± 1.4 ^a^	0.00	-	-

The different letters represent significant differences.

**Table 3:** Effect of the tested monoterpenes compared to the fungicide on the percentage of damping-off and disease incidence of bean plants under greenhouse conditions. Data are the average two locations.

Treatment	Damping-Off %			DI%
	Pre-Emergence	Post-Emergence	Survival	
Cuminaldehyde	37.7 ^b^ ± 0.23	0.0 ^e^ ± 0.00	63.3 ^b^ ± 0.72	22.2 ^d^ ± 1.03
Linalool	37.7 ^b^ ± 0.28	3.3 ^d^ ± 0.38	60.0 ^b^ ± 0.74	31.6 ^b^ ± 1.07
Carvone	23.4 ^c^ ± 0.19	13.3 ^a^ ± 0.42	63.3 ^b^ ± 0.77	26.3 ^c^ ± 1.08
Fungicide	10.0 ^d^ ± 0.17	6.67 ^c^ ± 0.32	83.3 ^a^ ± 0.79	20.0 ^e^ ± 0.99
Control	56.7 ^a^ ± 0.24	10.0 ^b^ ± 0.31	33.3 ^c^ ± 0.71	50.0 ^a^ ± 1.04
L.S.D	4.346	2.726	3.495	1.262

Statistical comparisons were made among treatments within a single column. The different letters represent significant differences using Fisher’s LSD test at *p ≤* 0.05.

**Table 4:** Effect of monoterpenes compared to the fungicide on the percentages of damping-off and disease incidence of *R. solani* in bean plants under field conditions in Kafrelshiekh and Gharbia governorates.

Treatment	Damping-Off %			DI%	% Efficacy
	Pre-Emergence	Post-Emergence	Survival		
Kafr Elsheikh governorate					
Cuminaldehyde	22.3 ^c^ ± 0.33	17.3 ^c^ ± 0.43	60.4 ^c^ ± 0.72	26.8 ^c^ ± 1.12	55.48 ± 2.23
Linalool	23.2 ^c^ ± 0.34	21.9 ^b^ ± 0.44	54.9 ^d^ ± 0.74	34.2 ^b^ ± 1.18	42.3 ± 2.29
Carvone	17.7 ^b^ ± 0.32	13.3 ^d^ ± 0.42	69.0 ^b^ ± 0.71	23.7 ^d^ ± 1.18	60.0 ± 2.28
Fungicide	12.0 ^a^ ± 0.35	7.7 ^e^ ± 0.43	80.3 ^a^ ± 0.77	18.9 ^e^ ± 1.17	68.1 ± 2.21
Control	34.8 ^d^ ± 0.38	34.3 ^a^ ± 0.41	30.9 ^e^ ± 0.78	59.3 ^a^ ± 1.21	0.00
L.S.D	2.214	2.328	2.973	3.298	--
Gharbia governorate					
Cuminaldehyde	24.6 ^b^ ± 0.34	18.2 ^c^ ± 0.45	57.2 ^c^ ± 0.75	28.2 ^c^ ± 1.19	54.3 ± 2.28
Linalool	28.2 ^b^ ± 0.37	23.2 ^b^ ± 0.43	48.6 ^d^ ± 0.78	36.4 ^b^ ± 1.21	41.0 ± 2.32
Carvone	19.3 ^c^ ± 0.31	12.1 ^d^ ± 0.47	68.6 ^b^ ± 0.74	21.3 ^d^ ± 1.19	65.5 ± 2.30
Fungicide	13.2 ^d^ ± 0.39	6.8 ^e^ ± 0.45	80.0 ^a^ ± 0.79	17.8 ^e^ ± 1.20	71.2 ± 2.28
Control	36.4 ^a^ ± 0.34	37.2 ^a^ ± 0.44	26.4 ^e^ ± 0.74	61.7 ^a^ ± 1.23	0.00
L.S.D	2.434	2.551	3.271	3.418	--

Statistical comparisons were made among treatments within a single column. The different letters represent significant differences using Fisher’s LSD test at *p ≤* 0.05.

**Table 5:** Effect of the tested monoterpenes compared to the fungicide on some growth parameters of bean plants under field conditions in Kafrelshiekh and Gharbia governorates.

Treatment	Growth Parameters			
	Plant Height (cm)	Branches No./Plant	Fresh Weight (g)/Plant	Dry Weight (g)/Plant
Kafr Elsheikh governorate				
Cuminaldehyde	47.4 ^b^ ± 1.21	5.2 ^b^ ± 0.74	79.3 ^b^ ± 2.10	10.4 ^a^ ± 0.98
Linalool	44.9 ^c^ ± 1.23	4.8 ^b^ ± 0.72	74.8 ^c^ ± 2.12	9.8 ^ab^ ± 0.94
Carvone	48.1 ^b^ ± 1.11	5.7 ^a^ ± 0.83	80.3 ^b^ ± 2.01	10.8 ^a^ ± 1.03
Fungicide	50.2 ^a^ ± 1.13	5.9 ^a^ ± 0.89	82.4 ^a^ ± 2.11	11.4 ^a^ ± 1.00
Control	34.5 ^d^ ± 1.17	3.2 ^c^ ± 0.92	41.1 ^d^ ± 1.99	5.3 ^c^ ± 1.01
L.S.D	1.438	0.423	2.327	0.612
Gharbia governorate				
Cuminaldehyde	45.8 ^b^ ± 1.23	4.8 ^b^ ± 0.71	77.1 ^b^ ± 2.14	10.0 ^b^ ± 0.98
Linalool	43.4 ^c^ ± 1.22	4.5 ^b^ ± 0.73	73.3 ^c^ ± 2.13	9.2 ^c^ ± 0.97
Carvone	47.3 ^b^ ± 1.17	5.5 ^a^ ± 0.80	78.9 ^b^ ± 2.12	10.3 ^b^ ± 1.11
Fungicide	48.3 ^a^ ± 1.15	5.7 ^a^ ± 0.88	81.2 ^a^ ± 2.17	11.0 ^a^ ± 1.10
Control	32.9 ^d^ ± 1.19	3.1 ^c^ ± 0.97	40.2 ^d^ ± 2.12	5.1 ^d^ ± 1.12
L.S.D	1.393	0.372	2.429	0.727

Statistical comparisons were made among treatments within a single column. The different letters represent significant differences using Fisher’s LSD test at *p ≤* 0.05.

**Table 6:** Effect of the tested monoterpenes compared to the fungicide on some yield parameters of bean plants under field conditions in Kafrelsheikh and Gharbia governorates.

Treatment	Yield Parameters			
	No. Pods/Plant	Pods Weight (g)/Plant	Production/Hectare (ton)	Percentage of Yield Increase
Kafr Elsheikh governorate				
Cuminaldehyde	17.6 ^b^ ± 1.23	19.13 ^ab^ ± 1.4	2.80 ^ab^ ± 0.21	35.7 ± 1.91
Linalool	15.3 ^c^ ± 1.33	17.51 ^c^ ± 1.32	2.61 ^b^ ± 0.23	31.3 ± 1.93
Carvone	18.7 ^b^ ± 1.24	20.11 ^a^ ± 1.33	2.88 ^a^ ± 0.21	38.9 ± 1.98
Fungicide	20.2 ^a^ ± 1.32	21.21 ^a^ ± 1.34	3.02 ^a^ ± 0.22	42.1 ± 1.94
Control	9.3 ^d^ ± 1.34	12.30 ^d^ ± 1.29	1.76 ^c^ ± 0.24	0.0
L.S.D	1.437	1.082	0.132	--
Gharbia governorate				
Cuminaldehyde	15.2 ^b^ ± 1.23	17.4 ^b^ ± 1.4	2.21 ^b^ ± 0.21	163.5 ± 1.91
Linalool	14.1 ^c^ ± 1.33	16.7 ^c^ ± 1.32	2.11^bc^ ± 0.23	134.7 ± 1.93
Carvone	17.2 ^a^ ± 1.24	18.4 ^b^ ± 1.33	2.64 ^b^ ± 0.21	215.4 ± 1.98
Fungicide	18.3 ^a^ ± 1.32	20. 1 ^a^ ± 1.34	3.07 ^a^ ± 0.22	266.7 ± 1.94
Control	8.8 ^d^ ± 1.34	11.4 ^d^ ± 1.32	0.83 ^d^ ± 0.24	0.00
L.S.D	1.548	1.082	0.178	--

Statistical comparisons were made among treatments within a single column. The different letters represent significant differences using Fisher’s LSD test at *p ≤* 0.05.

**Table 7:** Effect of the tested monoterpenes compared to the fungicide on activity of catalase, peroxidase, and polyphenoloxidae enzymes in bean plants under greenhouse conditions.

Treatment	Enzymatic Activities		
	CAT mM H_2_O_2_ g^−1^ FW Min^−1^	POX mM H_2_O_2_ g^−1^ FW Min^−1^	PPO µ mol/min^−1^ g^−1^ (FW)
Cuminaldehyde	32.4 ^a^ ± 2.21	0.871 ^a^ ± 0.08	0.143 ^b^ ± 0.02
Linalool	27.3 ^b^ ± 2.23	0.643 ^b^ ± 0.09	0.139 ^c^ ± 0.01
Carvone	33.2 ^a^ ± 2.32	0.989 ^a^ ± 0.08	0.155 ^a^ ± 0.03
Fungicide	21.3 ^c^ ± 2.22	0.497 ^c^ ± 0.07	0.117 ^d^ ± 0.02
Control	11.2 ^d^ ± 2.34	0.323 ^d^ ± 0.09	0.098 ^e^ ± 0.03
L.S.D	1.034	0.102	0.011

Statistical comparisons were made among treatments within a single column. The different letters represent significant differences using Fisher’s LSD test at *p ≤* 0.05.

## Data Availability

Not applicable.
